# *catena*-Poly[[dibromidozinc(II)]-μ-4-(3-pyrid­yl)-4*H*-1,2,4-triazole]

**DOI:** 10.1107/S1600536810026188

**Published:** 2010-07-14

**Authors:** Bin Ding, Hong-Ai Zou

**Affiliations:** a Tianjin Key Laboratory of Structure and Performance for Functional Molecules, Tianjin Normal University, Tianjin 300071, People’s Republic of China

## Abstract

The title complex, [ZnBr_2_(C_7_H_6_N_4_)]_*n*_, was formed under hydro­thermal conditions using the ligand 4-(3-pyrid­yl)-4*H*-1,2,4-triazole (*L*). The unique Zn^II^ ion is coordinated by one triazole N atom, one pyridine N atom and two Br atoms in a slightly distorted tetra­hedral coordination environment. Symmetry-related Zn^II^ ions are connected by bridging *L* ligands into chains parallel to [001] in which the Zn⋯Zn separation is 8.643 (7) Å. In the crystal structure, weak inter­molecular C—H⋯Br hydrogen bonds link the chains into a three-dimensional network.

## Related literature

For the preparation of the ligand used to synthesize the title compound, see: Gioia *et al.* (1988 ▶). For background literature on supra­molecular polymer chemistry, see: Lehn (1995 ▶); Ouahab (1997 ▶). For complexes incorporating 4-3-pyridyl-1,2,4-triazole ligands, see: Moulton & Zaworotko (2001 ▶); Pan *et al.* (2001 ▶); Prior & Rosseinsky (2001 ▶); Ma *et al.* (2001 ▶); Ding *et al.* (2006 ▶).
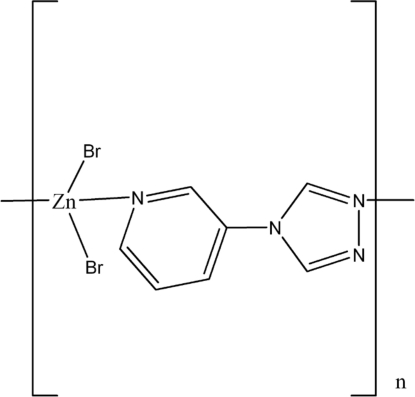


## Experimental

### 

#### Crystal data

[ZnBr_2_(C_7_H_6_N_4_)]
*M* *_r_* = 371.35
Monoclinic, 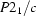

*a* = 6.787 (6) Å
*b* = 18.769 (15) Å
*c* = 8.643 (7) Å
β = 101.316 (11)°
*V* = 1079.6 (15) Å^3^
*Z* = 4
Mo *K*α radiation
μ = 9.64 mm^−1^
*T* = 293 K
0.18 × 0.12 × 0.06 mm

#### Data collection

Bruker APEXII diffractometer
Absorption correction: multi-scan (*SADABS*; Sheldrick, 1996 ▶) *T*_min_ = 0.522, *T*_max_ = 1.000
5681 measured reflections
1903 independent reflections
1510 reflections with *I* > 2σ(*I*)
*R*_int_ = 0.041

#### Refinement

*R*[*F* ^2^ > 2σ(*F* ^2^)] = 0.039
*wR*(*F* ^2^) = 0.090
*S* = 1.10
1903 reflections
128 parameters
H-atom parameters constrained
Δρ_max_ = 0.65 e Å^−3^
Δρ_min_ = −0.60 e Å^−3^

### 

Data collection: *APEX2* (Bruker, 2007 ▶); cell refinement: *SAINT* (Bruker, 2007 ▶); data reduction: *SAINT*; program(s) used to solve structure: *SHELXS97* (Sheldrick, 2008 ▶); program(s) used to refine structure: *SHELXL97* (Sheldrick, 2008 ▶); molecular graphics: *DIAMOND* (Brandenburg, 1999 ▶); software used to prepare material for publication: *publCIF* (Westrip, 2010 ▶).

## Supplementary Material

Crystal structure: contains datablocks global, I. DOI: 10.1107/S1600536810026188/lh5068sup1.cif

Structure factors: contains datablocks I. DOI: 10.1107/S1600536810026188/lh5068Isup2.hkl

Additional supplementary materials:  crystallographic information; 3D view; checkCIF report

## Figures and Tables

**Table 1:** Hydrogen-bond geometry (Å, °)

*D*—H⋯*A*	*D*—H	H⋯*A*	*D*⋯*A*	*D*—H⋯*A*
C7—H7⋯Br1^i^	0.93	2.92	3.711 (7)	145
C6—H6⋯Br2^ii^	0.93	2.93	3.779 (8)	153

Symmetry codes: (i) 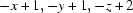; (ii) 
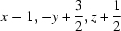
.

**Fig. 1.**
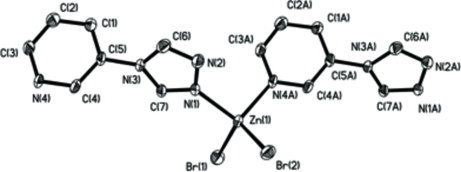
A view of the coordination around the ZnII ion of the title 1-D compound [symmetry code: (A) x, y, z - 1].

**Fig. 2.**
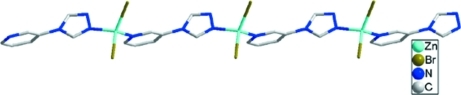
One-dimensional structure of the title compound

**Fig. 3.**
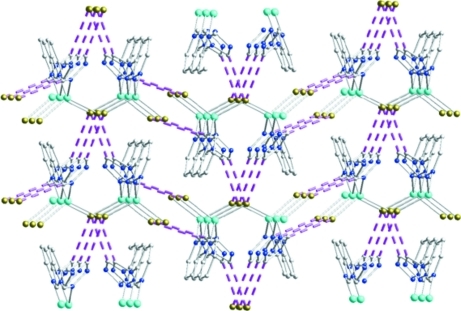
Part of the crystal structure of the title compound showing hydrogen bonds as dashed lines.

## supplementary crystallographic information

### Comment

Supramolecular polymer chemistry is a branch of modern science which is developing rapidly through the combination of polymer chemistry with supramolecular chemistry (Lehn, 1995; Ouahab, 1997). Recently, considerable efforts have been devoted to crystal engineering of supramolecular architecture sustained by coordination covalent bonding, hydrogen bonding or some molecular interaction and their combination. The compounds formed are of interest owing to their fascinating structural diversity and potential application in design of porous materials with novel inclusion or reactivity properties and in supramolecular devices such as sensors and indicators (Moulton & Zaworotko, 2001; Pan *et al.*, 2001; Prior & Rosseinsky, 2001; Ma *et al.*, 2001; Ding *et al.*, 2006). We report herein the crystal structure of the title complex.

A view of the coordination around the Zn^II^ ion of the title compound is shown in Fig. 1. The unique Zn^II^ ion is coordinated by one triazole nitrogen atom, one pyridine nitrogen atom and two bromine ligands in a slightly distorted tetrahedral coordination environment. Symmetry related Zn^II^ ions are connected by bridging *L* ligands to form one-dimensional chains (Fig. 2) in which the Zn···Zn separation is 8.643 (7) Å. In the crystal structure, weak intermolecular C—H···Br hydrogen bonds (Table 1) exist between *L* triazole rings and bromine atoms pairs of inversion related 1-D chains, which are further assembled through C—H···Br interactions to form a 3-D network (see Fig. 3).

### Experimental

The ligand *L* was prepared according to the previously reported literature methods (Gioia, *et al.*, 1988). A mixture of ZnBr_2_ (22.5 mg, 0.1 mmol), *L* (14.6 mg, 0.1 mmol) and water (10 ml) was stirred for 5 h and filtered. The filtrate was kept in a CaCl_2_ desiccator. Suitable single crystals for X-ray diffraction study were obtained after a few days, yield 23% (based on Zn(II) salts). Anal. Calc. for C_7_H_6_Br_2_N_4_Zn: C, 22.64%; H, 1.63%; N, 15.09%. Found: C, 22.75%; H, 1.87%; N, 15.14%. FT—IR (KBr): 3115 (w), 3050 (w), 2940(w), 1540(*s*), 1473(*m*), 1395(*m*), 1368(w), 1244(w), 1199(*s*), 1075(*s*), 1030(*s*), 978(w), 945(w), 869(*s*), 684(w), 640 (*s*), 489(*m*), 425 (w) cm_-1_.

### Refinement

H atoms were positioned geometrically and were allowed to ride on their parent C atoms with C—H = 0.93Å and *U*_iso_(H) = 1.2*U*_eq_(C).

### Crystal data

**Table:**

[ZnBr_2_(C_7_H_6_N_4_)]	*F*(000) = 704
*M**_r_* = 371.35	*D*_x_ = 2.285 Mg m^−^^3^
Monoclinic, *P*2_1_/*c*	Mo *K*α radiation, λ = 0.71073 Å
Hall symbol: -P 2ybc	Cell parameters from 1387 reflections
*a* = 6.787 (6) Å	θ = 2.6–24.1°
*b* = 18.769 (15) Å	µ = 9.64 mm^−^^1^
*c* = 8.643 (7) Å	*T* = 293 K
β = 101.316 (11)°	Block, colorless
*V* = 1079.6 (15) Å^3^	0.18 × 0.12 × 0.06 mm
*Z* = 4	

### Data collection

**Table:**

Bruker APEXII diffractometer	1903 independent reflections
Radiation source: fine-focus sealed tube	1510 reflections with *I* > 2σ(*I*)
graphite	*R*_int_ = 0.041
φ and ω scans	θ_max_ = 25.0°, θ_min_ = 2.2°
Absorption correction: multi-scan (*SADABS*; Sheldrick, 1996)	*h* = −7→8
*T*_min_ = 0.522, *T*_max_ = 1.000	*k* = −22→22
5681 measured reflections	*l* = −10→7

### Refinement

**Table:**

Refinement on *F*^2^	Secondary atom site location: difference Fourier map
Least-squares matrix: full	Hydrogen site location: inferred from neighbouring sites
*R*[*F*^2^ > 2σ(*F*^2^)] = 0.039	H-atom parameters constrained
*wR*(*F*^2^) = 0.090	*w* = 1/[σ^2^(*F*_o_^2^) + (0.0105*P*)^2^ + 4.1488*P*] where *P* = (*F*_o_^2^ + 2*F*_c_^2^)/3
*S* = 1.10	(Δ/σ)_max_ = 0.001
1903 reflections	Δρ_max_ = 0.65 e Å^−^^3^
128 parameters	Δρ_min_ = −0.60 e Å^−^^3^
0 restraints	Extinction correction: *SHELXL97* (Sheldrick, 2008)
Primary atom site location: structure-invariant direct methods	Extinction coefficient: 0.00010 (0)

### Special details

**Table:**

Geometry. All e.s.d.'s (except the e.s.d. in the dihedral angle between two l.s. planes) are estimated using the full covariance matrix. The cell e.s.d.'s are taken into account individually in the estimation of e.s.d.'s in distances, angles and torsion angles; correlations between e.s.d.'s in cell parameters are only used when they are defined by crystal symmetry. An approximate (isotropic) treatment of cell e.s.d.'s is used for estimating e.s.d.'s involving l.s. planes.
Refinement. Refinement of *F*^2^ against ALL reflections. The weighted *R*-factor *wR* and goodness of fit *S* are based on *F*^2^, conventional *R*-factors *R* are based on *F*, with *F* set to zero for negative *F*^2^. The threshold expression of *F*^2^ > σ(*F*^2^) is used only for calculating *R*-factors(gt) *etc*. and is not relevant to the choice of reflections for refinement. *R*-factors based on *F*^2^ are statistically about twice as large as those based on *F*, and *R*- factors based on ALL data will be even larger.

### Fractional atomic coordinates and isotropic or equivalent isotropic displacement parameters (Å^2^)

**Table:**

	*x*	*y*	*z*	*U*_iso_*/*U*_eq_	
Zn1	0.39470 (11)	0.63872 (4)	0.68138 (8)	0.0337 (2)	
Br1	0.61578 (12)	0.54147 (4)	0.76801 (9)	0.0506 (3)	
Br2	0.54852 (11)	0.74575 (4)	0.62834 (9)	0.0469 (2)	
N1	0.2184 (8)	0.6476 (3)	0.8432 (5)	0.0334 (12)	
N2	0.0687 (8)	0.6990 (3)	0.8251 (6)	0.0435 (14)	
N3	0.0872 (8)	0.6435 (3)	1.0539 (5)	0.0324 (12)	
N4	0.1730 (8)	0.6178 (3)	1.4833 (5)	0.0339 (12)	
C1	−0.1373 (10)	0.5977 (4)	1.2212 (8)	0.0419 (17)	
H1	−0.2388	0.5907	1.1332	0.050*	
C2	−0.1642 (11)	0.5823 (4)	1.3737 (8)	0.0513 (19)	
H2	−0.2868	0.5653	1.3907	0.062*	
C3	−0.0079 (10)	0.5926 (4)	1.4974 (8)	0.0415 (17)	
H3	−0.0279	0.5815	1.5980	0.050*	
C4	0.2014 (10)	0.6340 (3)	1.3381 (7)	0.0373 (15)	
H4	0.3248	0.6520	1.3251	0.045*	
C5	0.0513 (10)	0.6243 (3)	1.2085 (7)	0.0333 (15)	
C6	−0.0101 (11)	0.6954 (4)	0.9528 (8)	0.0451 (17)	
H6	−0.1155	0.7235	0.9722	0.054*	
C7	0.2253 (9)	0.6162 (3)	0.9792 (7)	0.0322 (14)	
H7	0.3142	0.5799	1.0188	0.039*	

### Atomic displacement parameters (Å^2^)

**Table:**

	*U*^11^	*U*^22^	*U*^33^	*U*^12^	*U*^13^	*U*^23^
Zn1	0.0344 (4)	0.0455 (5)	0.0238 (4)	−0.0007 (3)	0.0122 (3)	0.0026 (3)
Br1	0.0527 (5)	0.0476 (4)	0.0534 (5)	0.0094 (4)	0.0153 (4)	0.0096 (3)
Br2	0.0419 (4)	0.0489 (4)	0.0529 (5)	−0.0042 (3)	0.0168 (3)	0.0093 (3)
N1	0.033 (3)	0.047 (3)	0.021 (2)	0.002 (3)	0.007 (2)	0.003 (2)
N2	0.042 (4)	0.055 (4)	0.036 (3)	0.011 (3)	0.015 (3)	0.012 (3)
N3	0.033 (3)	0.045 (3)	0.022 (2)	0.000 (2)	0.010 (2)	0.002 (2)
N4	0.041 (3)	0.042 (3)	0.023 (3)	−0.001 (3)	0.015 (2)	0.002 (2)
C1	0.038 (4)	0.057 (4)	0.031 (3)	−0.006 (3)	0.009 (3)	−0.002 (3)
C2	0.044 (5)	0.067 (5)	0.045 (4)	−0.014 (4)	0.014 (4)	0.002 (4)
C3	0.044 (4)	0.054 (4)	0.029 (3)	−0.010 (3)	0.013 (3)	0.006 (3)
C4	0.041 (4)	0.047 (4)	0.029 (3)	−0.002 (3)	0.019 (3)	0.002 (3)
C5	0.042 (4)	0.039 (3)	0.021 (3)	−0.002 (3)	0.013 (3)	−0.003 (3)
C6	0.041 (4)	0.052 (4)	0.046 (4)	0.011 (3)	0.019 (3)	0.006 (3)
C7	0.036 (4)	0.038 (3)	0.023 (3)	0.003 (3)	0.008 (3)	0.000 (3)

### Geometric parameters (Å, °)

**Table:**

Zn1—N1	2.018 (5)	N4—Zn1^ii^	2.083 (5)
Zn1—N4^i^	2.083 (5)	C1—C2	1.396 (9)
Zn1—Br2	2.3502 (18)	C1—C5	1.397 (9)
Zn1—Br1	2.3880 (17)	C1—H1	0.9300
N1—C7	1.308 (7)	C2—C3	1.364 (9)
N1—N2	1.388 (7)	C2—H2	0.9300
N2—C6	1.319 (8)	C3—H3	0.9300
N3—C7	1.339 (8)	C4—C5	1.370 (9)
N3—C6	1.386 (8)	C4—H4	0.9300
N3—C5	1.450 (7)	C6—H6	0.9300
N4—C4	1.341 (7)	C7—H7	0.9300
N4—C3	1.343 (8)		
			
N1—Zn1—N4^i^	98.9 (2)	C3—C2—C1	119.0 (6)
N1—Zn1—Br2	114.26 (15)	C3—C2—H2	120.5
N4^i^—Zn1—Br2	106.11 (14)	C1—C2—H2	120.5
N1—Zn1—Br1	105.51 (15)	N4—C3—C2	124.2 (6)
N4^i^—Zn1—Br1	114.96 (15)	N4—C3—H3	117.9
Br2—Zn1—Br1	116.02 (7)	C2—C3—H3	117.9
C7—N1—N2	108.1 (5)	N4—C4—C5	120.9 (6)
C7—N1—Zn1	131.6 (4)	N4—C4—H4	119.5
N2—N1—Zn1	120.0 (4)	C5—C4—H4	119.5
C6—N2—N1	106.1 (5)	C4—C5—C1	121.9 (6)
C7—N3—C6	104.9 (5)	C4—C5—N3	119.2 (6)
C7—N3—C5	127.6 (5)	C1—C5—N3	118.9 (5)
C6—N3—C5	127.6 (5)	N2—C6—N3	110.0 (6)
C4—N4—C3	117.8 (6)	N2—C6—H6	125.0
C4—N4—Zn1^ii^	120.9 (4)	N3—C6—H6	125.0
C3—N4—Zn1^ii^	121.1 (4)	N1—C7—N3	110.9 (6)
C2—C1—C5	116.1 (6)	N1—C7—H7	124.6
C2—C1—H1	122.0	N3—C7—H7	124.6
C5—C1—H1	122.0		
			
N4^i^—Zn1—N1—C7	127.0 (6)	N4—C4—C5—N3	179.0 (5)
Br2—Zn1—N1—C7	−120.8 (5)	C2—C1—C5—C4	0.6 (10)
Br1—Zn1—N1—C7	7.9 (6)	C2—C1—C5—N3	−178.1 (6)
N4^i^—Zn1—N1—N2	−59.6 (5)	C7—N3—C5—C4	61.5 (9)
Br2—Zn1—N1—N2	52.7 (5)	C6—N3—C5—C4	−116.6 (7)
Br1—Zn1—N1—N2	−178.7 (4)	C7—N3—C5—C1	−119.8 (7)
C7—N1—N2—C6	−0.7 (7)	C6—N3—C5—C1	62.1 (9)
Zn1—N1—N2—C6	−175.6 (4)	N1—N2—C6—N3	0.8 (8)
C5—C1—C2—C3	−1.1 (10)	C7—N3—C6—N2	−0.6 (7)
C4—N4—C3—C2	0.1 (10)	C5—N3—C6—N2	177.8 (6)
Zn1^ii^—N4—C3—C2	175.4 (6)	N2—N1—C7—N3	0.3 (7)
C1—C2—C3—N4	0.8 (11)	Zn1—N1—C7—N3	174.4 (4)
C3—N4—C4—C5	−0.7 (9)	C6—N3—C7—N1	0.2 (7)
Zn1^ii^—N4—C4—C5	−176.0 (5)	C5—N3—C7—N1	−178.3 (6)
N4—C4—C5—C1	0.4 (10)		

Symmetry codes: (i) *x*, *y*, *z*−1; (ii) *x*, *y*, *z*+1.

### Hydrogen-bond geometry (Å, °)

**Table:**

*D*—H···*A*	*D*—H	H···*A*	*D*···*A*	*D*—H···*A*
C7—H7···Br1^iii^	0.93	2.92	3.711 (7)	145
C6—H6···Br2^iv^	0.93	2.93	3.779 (8)	153

Symmetry codes: (iii) −*x*+1, −*y*+1, −*z*+2; (iv) *x*−1, −*y*+3/2, *z*+1/2.
